# CD8 Effector T Cells Function Synergistically With Broadly Neutralizing Antibodies to Enhance Suppression of HIV Infection

**DOI:** 10.3389/fimmu.2021.708355

**Published:** 2021-07-29

**Authors:** Rebecca T. Veenhuis, Caroline C. Garliss, Justin R. Bailey, Joel N. Blankson

**Affiliations:** ^1^Department of Molecular and Comparative Pathobiology, Johns Hopkins Medicine, Baltimore, MD, United States; ^2^Department of Medicine, Johns Hopkins Medicine, Baltimore, MD, United States

**Keywords:** HIV, CD8 lymphocytes +, broadly neutralising antibodies, synergy, elite controllers, elite suppressors

## Abstract

HIV-specific CD8 T cells and broadly neutralizing antibodies (bNAbs) both contribute to the control of viremia, but in most cases, neither can completely suppress viral replication. To date, therapeutic vaccines have not been successful in eliciting HIV-specific CD8 T cell or bNAb responses that are capable of preventing long-term viral rebound upon ART cessation. These challenges suggest that a combinatorial approach that harnesses both bNAbs and CD8 T cell responses may be necessary for long term control of viral replication. In this study we demonstrate a synergistic interaction between CD8 T cells and bNAbs using an *in vitro* model. Our data suggest that this combinatorial approach is very effective at suppressing viral replication *in vitro* and should be considered in future therapeutic studies.

## Introduction

There are more than 37 million people worldwide infected with human immunodeficiency virus (HIV). Although access to antiretroviral therapy (ART) has reduced HIV-related morbidity and mortality, it is not a cure. A vaccine or cure strategy is desperately needed to end the requirement for life-long ART.

HIV infection is characterized by high levels of plasma viremia that can be controlled, to varying degrees, by virus-specific immune responses. There are several lines of evidence that CD8 T cells contribute to the control of HIV replication. There is a temporal association between the emergence of HIV-specific CD8 T cells and the decline of viremia in primary infection (1, 2). There is an overrepresentation of certain Class I MHC alleles in patients known as elite suppressors (ES) or viremic controllers (VC) who control viral replication to low or undetectable levels without ART (3, 4). Many of these subjects have more potent HIV-specific CD8 T cell responses than patients known as chronic progressors (CP) who do not control viral replication without ART (4–8). Further, in the simian immunodeficiency virus (SIV) macaque model of HIV infection, the depletion of CD8 lymphocytes leads to rebound of SIV viremia in animals that had previously controlled viremia (9). Additionally, the reappearance of SIV-specific CD8 T cells coincides with reestablishment of viral control (10). Therefore, it is clear that optimal CD8 T cell responses to HIV are essential for viral control.

Broadly neutralizing antibodies (bNAbs) can also contribute to HIV control and have dual functionality; the variable regions neutralize the virus, whereas the constant domains can engage Fc receptors on effector cells of the immune system (11). The administration of bNAbs immediately after infection has been shown to prevent infection and seeding of the latent HIV reservoir (12). Additionally, in human trials, CD4 binding-site (CD4bs) Abs have a transient effect on viral load in individuals who are not on ART, and administration of bNAbs during analytical treatment interruption (ATI) can delay rebound of the virus (13–15).

The use of vaccines or other therapeutic strategies to boost immune responses to the virus may eventually lead to long term HIV remission. Therapeutic vaccines aim to either improve the functional capacity of the host CD8 response to kill infected CD4 T cells or increase the potency of circulating antibodies able to neutralize circulating viruses. Thus far, therapeutic vaccines have proven to be unsuccessful, as previous vaccine strategies have shown some induction of CD8 T cells or neutralizing antibodies, but they have not led to long term control of viral replication when ART is discontinued (16, 17). The challenges that have arisen in the development of such a vaccine suggest that a combinatorial approach may be necessary to harness both neutralizing antibodies and sub optimal CD8 T cell responses to suppress virus replication.

The goal of our study was to develop an *in vitro* model that assessed whether suboptimal CD8 T cell responses and bNAb treatment function synergistically or independently to suppress HIV infection. Thus, we designed experiments to interrogate how viral replication proceeds in the presence of CD8 T cells and bNAbs, separately or in combination. Our results have implications for HIV therapeutic and cure strategies.

## Methods

### Subjects

Blood samples from HIV-negative and HIV-positive donors were obtained with written informed consent and subsequently handled in accordance with protocols approved by the Johns Hopkins University IRB. HIV controllers are made up of two different classes of subjects. An elite suppressor (ES) refers to a subject who has maintained undetectable viral loads in the absence of ART (18). A viremic controller (VC) refers to a patient who has maintained viral loads below 1000 copies/ml in the absence of ART (19).

### NL4.3-Delta-Env-GFP X4 Virus

A single round X4 tropic enveloped NL4-3 virus with GFP engineered into env was generated as previously reported (20). In brief, NL4-3 delta env backbone and a separate X4 envelop plasmid were transfected into 293T cells using lipofectimine following manufacturer’s recommendations. Virus supernatants were collected 72h post transfection an ultracentrifuged to concentrate and purify virus. Virus was reconstituted in R10 ON at 4.C and then assessed by p24 ELISA (Perkin Elmer) to determine concentration. All virus stocks were test on healthy donor CD4s to determine effective concentrations to allow for 20-30% GFP positive CD4 T cells. The X4 envelop was derived from the HIV-IIIB virus and considered to be highly sensitive to neutralization, tier 1B (21–23).

### bNAb IC50 Calculations

**Figure 1:**
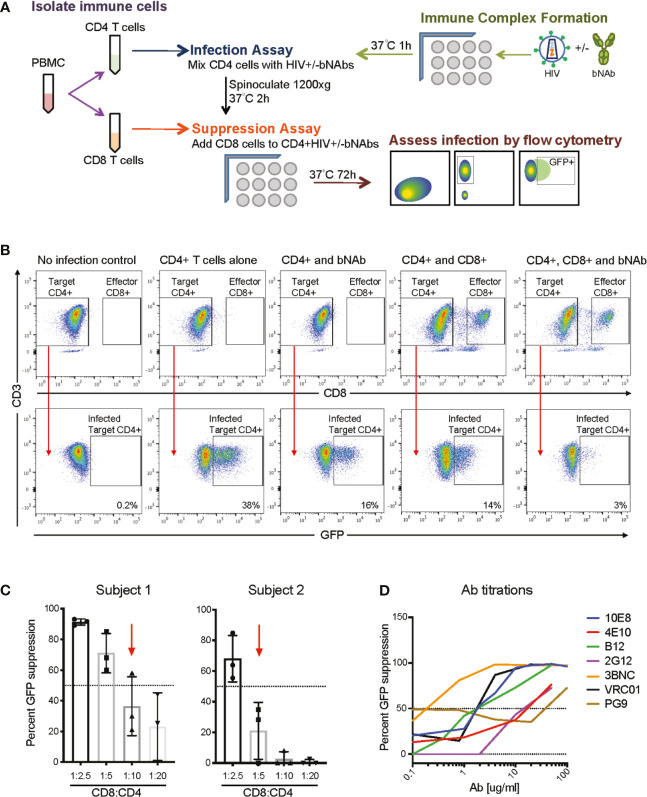
Overview of bNAb+CD8 suppression assay experimental scheme. **(A)** A schematic of the overall assay design and analysis. **(B)** Representative FACS plots demonstrating CD4 gating and GFP+ gating in the negative control (no infection), positive control (CD4 T cells alone), and experimental conditions (CD4 and bNAb; CD4 and CD8; CD4, CD8 and bNAb), GFP+ percentages shown. **(C)** Displays the E:T ratios used for two representative subjects to show how E:T ratios were chosen for each individual studied, CD8:CD4 ratios which resulted in less than 60% GFP suppression were used for suppression and Bliss analyses. **(D)** Representative data of bNAb titrations completed to calculate IC50 concentrations for each bNAb to be used in the suppression assay.

All broadly Neutralizing Antibodies (bNAbs) were obtained from the NIH AIDs reagent bank, CD4bs: 3BNC117 (24, 25), VRC01 (26), b12 (27–30); V1-V2: PG9 (31); V3: 2G12 (32–36); MPER: 10E8 (37), 4E10 (38). PBMCs were collected from healthy donor whole blood after Ficoll-Paque Plus gradient centrifugation (GE Healthcare Life Sciences, Baltimore, MD) and CD4+ T cells were negatively selected (CD4^+^ T cell isolation kit, Miltenyi). Immune complex assays were setup as described in detail below. In brief, 100ng of p24/100,000 cells of NL4.3-delta-Env+X4 virus was incubated with a titration of each bNAb (**Figure 1C** and **Supplemental 1**). Immune complexes were allowed to form for 1 hr at 37°C and then added to CD4 T cells in triplicate. CD4s were then spinoculated at 1200 x*g* for 2h at 37°C (39), cells were reconstituted in 200ul of fresh media and then incubated at 37°C for 72h. All samples were then assessed for GFP expression by flow cytometry. IC50 Ab concentrations were calculated when 50% GFP expression was suppressed by the added bNAb (**Supplemental Table 2**). Each antibody was titrated in isolation with concentrations starting at 100μg/mL to as low as 0.00001μg/mL (**Supplemental Figure 1**), in an attempt to create “S” shaped curves. In some cases we were unable to achieve saturation on the high end of the curve due to the amount of bNAbs available. Additionally, some bNAbs were titrated on multiple donors due to variability in GFP suppression. IC50s were determined based on the average IC50 for each donor (b12, 10E8, 4E10, 2G12, **Supplemental Figure 1**). All calculated IC50s are listed in **Supplemental Table 2**, IC50 concentrations were used in all CD8 suppression assays.

### Immune Complex, CD4 T Cell Infection, and CD8 T Cell Suppression Assay

See **Figure 1A** for a detailed schematic of assay setup. Immune complex and CD8 T cell suppression assays were performed as previously described (40–42). In brief, 40ul of NL4-3deltaEnv+X4 virus (100ng of p24) was added to 40ul of the calculated IC50 of each bNAb, **Supplemental Table 2**. All neutralization assays were setup in independent wells, 15 wells per bNAb, 3 wells per effector-to-target (E:T) ratio. This was done so that triplicate CD4 infections could be completed per immune complex and E:T condition, and allowed for the assessment of variability in GFP suppression for each condition. Immune complexes were allowed to form for 1-2 h at 37°C prior to addition to CD4 T cells. During the immune complex assay incubation, PBMCs were isolated from patient blood, CD8 T cells were positively selected (CD8 T cell Isolation kit, Miltenyi), and CD4 T cells were isolated by negative selection (CD4 T cell isolation kit, Miltenyi). The purity of the isolated CD4 and CD8 T cell populations was routinely 90-95%. 20ul of isolated CD4 T cells (100,000 cells per well) were then added to the immune complex wells for a total volume of 100ul. The CD4 T cells were infected by spinoculation at 1,200 x*g* for 2h at 37°C. Post spinoculation, infected CD4 T cells were co-cultured with autologous CD8 T cells at multiple E:T ratios (see **Supplemental Figure 3** for details on each subject’s ratios) for 3 days at 37°C. Flow cytometry was then performed to assess the percentage of GFP+ CD4 T cells at day 3. All samples were gated on singlets (FSC A *vs* FSC H) and then on lymphocyte sized cells (FSC A *vs* SSC A), data not shown. CD4 T cells were determined by gating CD3+ (clone UCHT1) and CD8– (clone RPA-T8) cells (**Figure 1B**), GFP positive cells were determined by gating CD3+/CD8-/GFP+ cells, an uninfected control was used to set GFP+ gating (**Figure 1B**). All samples were run on a BD LSR Fortessa and analysis was completed using Flow Jo.

Percent GFP suppression calculations were completed using the following formula: %GFP suppression = 100-[(%GFP+ CD4_HIV+treatment_ ÷ %GFP+ CD4_HIVonly_)*100] see **Supplemental Table 1** for examples. bNAb concentrations resulting in 50% GFP suppression (bNAb_IC50_) or CD8:CD4 ratios in which 60% or less GFP suppression occurred (CD8_IC60_) were used for the analysis of GFP suppression with bNAb_IC50_ or CD8_IC60_ alone *vs* GFP suppression with bNAb_IC50_+CD8_IC60_, and in Bliss calculations. Additionally, we completed analysis to assess whether less potent CD8 function, defined as the E:T ratio resulting in 0-40% suppression by CD8 T cells (CD8_IC40_), could be rescued when bNAbs were included. To select CD8_IC40_ E:T ratios for each subject, we chose the E:T ratio from the CD8-only suppression experiments which resulted in the least GFP suppression of the ratios tested, or the highest E:T ratio to result in zero suppression (**Supplemental Figure 1**, blue arrows). We compared GFP suppression by CD8_IC40_ or bNAb_IC50_ alone to GFP suppression by bNAb_IC50_+CD8_IC40_. Bliss calculations were also completed for the same conditions.

### Statistics and Calculations

Statistical analyses comparing multiple groups were performed using a 1-way ANOVA with Tukey’s multiple comparison test. The Bliss independence model (43) was used to predict combined suppression of bNAb_IC50_ and CD8_IC60_ as previously described (44, 45). Formulas and example calculations are listed in **Supplemental Table 1**. In brief, experimental fraction unaffected (F_un_) values were calculated by dividing the percent GFP^+^ cells in the combined condition (CD8_IC60_+bNAb_IC50_) by the percentage of GFP^+^ cells in the CD4+virus only well. Bliss F_un_, the predicted value if the treatments are working independently, is calculated by multiplying the experimental F_un_ for each individual treatment, bNAb_IC50_ only and CD8_IC60_ only. The calculated Bliss F_un_ value is than compared to the experimental F_un_ value calculated for the combined treatment. If Bliss F_un_ = experimental F_un_, suggests independence; if Bliss F_un_ > experimental F_un_, suggests synergy; if Bliss F_un_ < experimental F_un_, suggests antagonism. Experimental F_un_ and Bliss F_un_ comparisons were completed using paired T tests. *P* values of less than 0.05 were considered significant.

### Study Approval

This study was approved by the Johns Hopkins University Institutional Review Board. Informed written consent was obtained from both subjects prior to enrollment into the study.

## Results

### Development of an Assay to Measure the Effects of CD8 Effector T Cells and bNAbs on HIV Infection and Replication

We developed a combination assay that uses autologous CD8 T cells from HIV controllers to suppress a heterologous infection of autologous CD4 T cells by virus that has been previously allowed to form immune complexes with bNAbs (**Figure 1A**). The goal of the assay was to determine whether the addition of bNAbs could enhance viral suppression by suboptimal CD8 T cell responses. The CD8 component of the suppression assay has been previously described (40–42), as have neutralization assays using bNAbs (46). This is, to our knowledge, the first report of CD8s and bNAbs being tested in combination in this assay. All results were calculated based on a percent of infected (GFP positive) CD4 T cells, as gated based on negative (CD4 T cells only) and positive (CD4 T cells + virus only) controls (**Figure 1B** and **Supplemental Table 1**). Percent viral suppression was measured by a loss of GFP signal when CD8 T cells, bNAbs or both were present during the infection, as shown in **Figure 1B**. CD8:CD4 T cell ratios that resulted in 60% GFP suppression or less (CD8_IC60_) were used for all analyses to model the effect of the suboptimal CD8 T cell responses seen in chronic progressors (CP) on ART (**Figure 1C** and **Supplemental Figure 1**). Additionally, using CD8 T cell responses that only suppressed up to 60% of the GFP signal allowed us to detect any increase in suppression when bNAbs were also added into the culture. Given different potency of CD8 T cells from different donors, optimal CD8:CD4 T cell effector:target (E:T) ratios differed for different donors, ranging from 1:2 to 1:20. bNAbs were used at concentrations based on calculated IC50s (**Figure 1D** and **Supplemental Figure 2**) as listed in **Supplemental Table 2**. IC50s were used so that only 50% of the GFP signal would be suppressed in the Ab-only controls, which allowed for any increase in suppression to be detected when autologous CD8 T cells were also added into the culture. This assay allows us to measure the suppressive effect of autologous CD8 T cells alone, bNAbs alone, or both in combination to determine whether the two arms of the adaptive immune system are working synergistically, independently or antagonistically using the Bliss independence model (43).

### Combination of bNAbs and Autologous CD8 T Cells Enhances Heterologous Suppression

**Figure 2:**
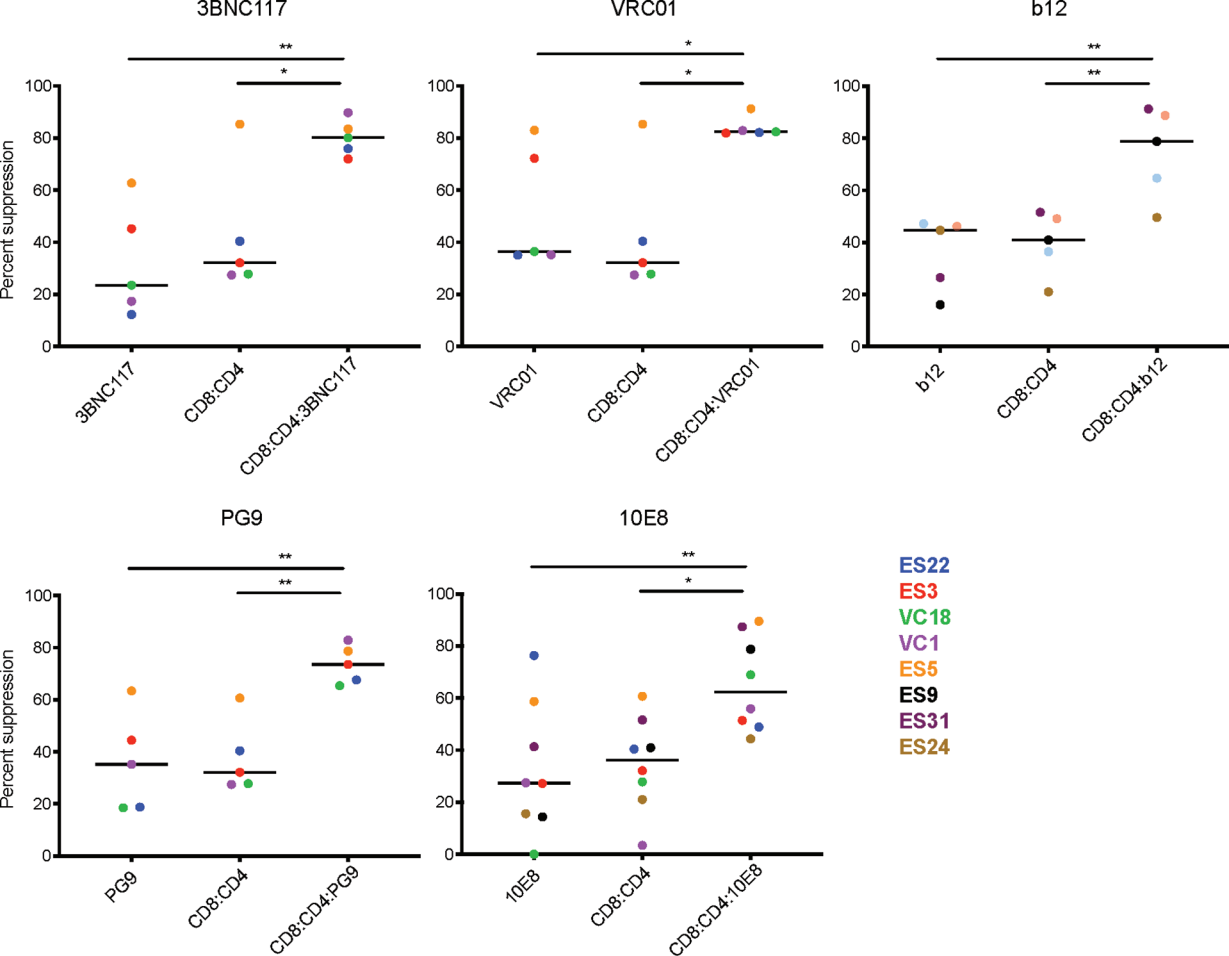
Combination of bNAbs and autologous CD8 T cells enhances heterologous suppression. CD4 T cells from 6 ES and 2 VC were infected with GFP-NL4.3deltaEnv_X4 with and without bNAb_IC50_ and then co-cultured with multiple CD8:CD4 ratios. The E:T which resulted in 60% or less of suppression was used to compare the levels of suppression in bNAb only well, CD8 only and CD8+bNAb wells. The combination of any CD8_IC60_+bNAb_IC50_ lead to enhanced suppression of HIV infection in all assays. Each data point displays the average of three technical replicates performed for each condition. The line indicates the median, each colored point represents data from one subject, and the value is calculated by averaging three technical replicates within the experiment. Statistical analysis was performed using a 1-way ANOVA with Tukey’s multiple comparison test, *p < 0.05 and **p < 0.01.

We measured CD8 T cell and bNAb suppression using CD4 and CD8 T cells isolated from 8 subjects with natural control of HIV infection (6 ES and 2 VC) and 5 different bNAbs that target the CD4 binding site (CD4bs), the V1-V2, or the membrane-proximal external region (MPER) of HIV Env (CD4bs: 3BNC117, VRC01, b12; V1-V2: PG9; MPER: 10E8). We found that for every bNAb tested, the addition of both CD8 T cells and bNAbs led to a significant enhancement in suppression of viral replication relative to either CD8 T cells or bNAbs alone (**Figure 2**). Notably, although bNAb concentrations and E:T ratios were optimized to result in 50% or up to 60% suppression, respectively, suppression by bNAbs or CD8s alone varied somewhat when T cells from different subjects were used in this experiment. Suppression by 3BNC117, VRC01, and 10E8 bNAbs alone was particularly variable, ranging from 0-80% suppression.

Despite the variability observed in bNAb only and CD8 T cell only suppression, there was still a significant difference when comparing CD8 T cell only or bNAb only to CD8+bNAb combinations. Suppression assays using CD4bs bNAbs, such as 3BNC117, VRC01 and b12, combined with autologous CD8 T cells resulted in median suppression values of 80%, 82%, and 78%, respectively, compared to bNAb or CD8 T cells alone conditions, which resulted in 25-42% suppression. Suppression assays using V1-V2 specific bNAbs or MPER bNAbs, such as PG9 and 10E8, combined with autologous CD8 T cells resulted in median suppression values of 73% and 62% suppression, respectively, compared to bNAb or CD8 T cell alone conditions that resulted in 27-35% suppression. In all cases the combination treatment median was more than twice that of the individual treatment medians, with p values ranging from 0.04 to 0.002. Additionally, due to limited availability, we tested the bNAbs 4E10 (MPER) and 2G12 (V3 glycan) using cells from a single subject and found a similar trend (**Supplemental Figure 3**). To assess the reproducibility of the assay, select subjects were tested in two to three independent experiments using the same bNAb_IC50_ concentrations, and E:T ratios selected to achieve 60% or less suppression by CD8 T cells alone (**Supplemental Figure 4**). We observed that in every case the addition of bNAb and CD8 T cells in combination led to an increase in suppression of viral replication despite some variability in the bNAb or CD8 T cell conditions. Taken together, the data presented here suggest that the combination of CD8 T cells and bNAb is significantly more effective at suppressing viral replication than CD8 T cells or bNAb alone.

### CD4bs bNAbs and CD8 T Cells Act Synergistically

**Figure 3:**
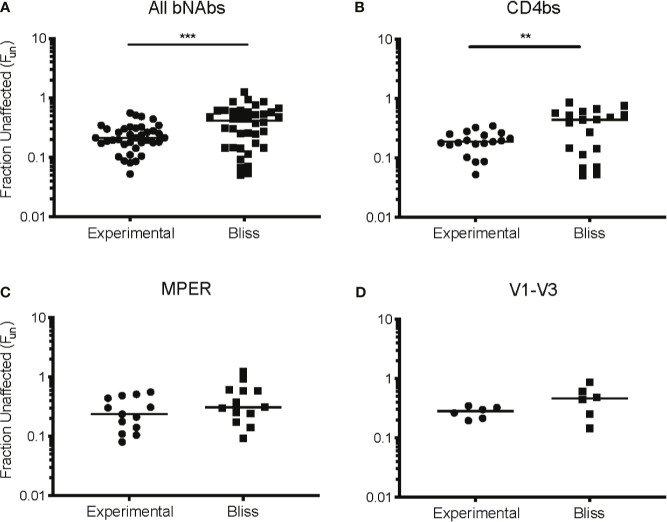
CD4 binding site bNAbs and CD8 T cells act synergistically to suppress HIV replication. Experimental F_un_ values from **(A)** all CD8+bNAb experiments, n = 38, **(B)** all CD4bs CD8+bNAb experiments, n = 19, **(C)** all MPER CD8+bNAb experiments, n = 13, and **(D)** all V1-V3 CD8+bNAb experiments, n = 6, were combined and compared to the predicted Bliss F_un_ values for the same conditions. Each Bliss or Fun data point is calculated from the average of three technical replicates performed for each condition. The line indicates median and each point indicates an independent experiment or predicated value. Statistical analysis was performed using paired t tests, **p = 0.005 and ***p = 0.0002.

**Figure 4:**
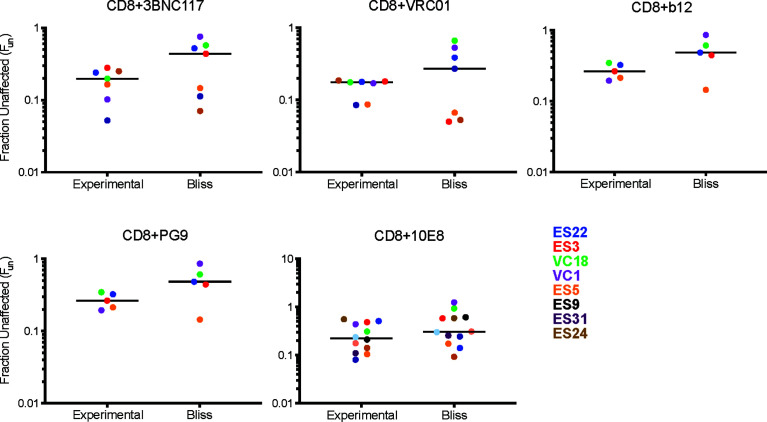
Individual bNAbs and CD8 T cells act independently to suppress HIV replication. Experimental F_un_ values from each individual bNAb were compared to the predicated Bliss F_un_ values. Each BLISS or Fun data point is calculated from the average of three technical replicates performed for each condition. The line indicates median and each point indicates an independent experiment or predicated value, one color per subject. Statistical analysis was performed using paired t tests.

We next investigated the interaction between CD8 T cell suppression and bNAb suppression using the Bliss independence model (43) (**Figure 3** and **Supplemental Table 1**). This model can be used to identify synergy or antagonism between inhibitors under the assumption that the inhibitors have independent binding sites and independent mechanisms of action. This assumption was appropriate for these experiments since bNAbs primarily inhibit viral entry, whereas CD8 effector T cells kill already-infected cells. Fraction unaffected (F_un_) values of all CD8+bNAb combinations tested (experimental) were lower than values calculated using the Bliss independence model from suppression by the same bNAbs and CD8s tested individually, indicating that CD8 T cells and bNAbs were functioning synergistically (**Figure 3A**, p=0.0002). Additionally, we analyzed these data broken down by bNAb class (CD4bs, MPER, and V1-V3 region). As when all bNAbs were analyzed together, we found that experimental F_un_ values were significantly lower than Bliss F_un_ values for the CD4bs class of bNAbs, indicating a synergistic interaction between CD8 T cells and CD4bs bNAbs (**Figure 3B**, p=0.005). There was also a trend toward lower experimental F_un_ values relative to Bliss calculated F_un_ values for MPER bNAbs and CD8 T cell and V1-V3 bNAbs and CD8 T cell combinations, although these differences were not statistically significant (**Figures 3C, D**), possibly due to the smaller number of antibodies tested in these categories. Finally, we analyzed experimental *vs*. Bliss comparisons for CD8 T cells +3BNC117, VRC01, b12, PG9, or 10E8 bNAbs individually (**Figure 4**). There was a trend towards lower experimental *vs*. Bliss F_un_ values for every individual bNAb, demonstrating that they all behaved similarly in combination with CD8 T cells. Taken together, these data demonstrate that CD4bs bNAbs and CD8 T cells act synergistically to inhibit HIV infection. There was no evidence of antagonism between any bNAb and CD8 T cells.

### bNAbs Act Synergistically With CD8 T Cells at Lower E:T Ratios

Next, we sought to determine if the addition of bNAb_IC50_ to CD8 T cells at E:T ratios with low CD8 suppression, defined as 0-40% GFP suppression (CD8_IC40_), would also result in enhanced GFP suppression. We found that for every bNAb tested, the combination of both CD8_IC40_ and bNAb_IC50_ led to an enhancement in suppression of viral replication relative to either CD8_IC40_ or bNAb_IC50_ alone (**Supplemental Figure 5**). However, the observed enhancements were no longer significant, unlike CD8_IC60_ E:T ratios in combination with bNAb_IC50_ (**Figure 2**).

Additionally, we investigated the interaction between lower suppression CD8 T cell ratios and bNAb_IC50_ suppression using the Bliss independence model (21) (**Supplemental Figure 6** and **Supplemental Table 1**). Fraction unaffected (F_un_) values of all CD8_IC40_+bNAb_IC50_ combinations tested (experimental) were lower than predicted values calculated using the Bliss model, indicating that CD8 T cells and bNAbs were functioning synergistically even with very low CD8:CD4 T cell ratios (**Supplemental Figure 6A**, p=0.005). Additionally, we analyzed these data broken down by bNAb class (CD4bs, MPER, and V1-V3 region). There was also a trend toward lower experimental F_un_ values relative to Bliss calculated F_un_ values for the CD4bs class of bNAbs (p=0.054, **Supplemental Figure 6B**) suggesting that with a larger sample size, CD4bs bNAbs and lower frequencies of CD8 T cells may act synergistically. Inhibition by MPER bNAbs or V1-3 bNAbs in combination with lower frequencies of CD8 T cells were not significantly different from values calculated using the Bliss model (**Supplemental Figures 6C, D**) as observed with CD8_IC60_+bNAb_IC50_ combinations (**Figure 4**).

## Discussion

In this study, we investigated the direct interaction between the suppressive capacity of CD8 T cells and neutralization by bNAbs in HIV infection. CD8 T cell receptors recognize peptides presented on MHC class I molecules and kill infected cells, whereas bNAbs recognize various epitopes on the virus envelope protein to prevent viral entry into CD4 T cells. Therefore, we asked whether the two different arms of the adaptive immune system could work synergistically to inhibit viral infection and replication. We observed a significant enhancement in viral suppression when autologous CD8s were used in combination with bNAbs. Additionally, we used the Bliss model to formally assess the interaction between the effector cells and bNAbs, demonstrating that bNAbs worked synergistically with CD8 effector T cells to suppress infection. These data suggest that a combination of CD4bs bNAbs and CD8 T cell responses could lead to enhanced suppression of HIV infection.

Early clinical trials identified several challenges for bNAbs as a therapeutic agent. These challenges included transient suppression of viremia, even at high doses (30–40 mg/kg), frequent emergence of resistance in rebound variants, suboptimal efficacy in preventing cell-to-cell viral transmission, and unclear effects on the cell-associated HIV-1 reservoir (47). However, despite these initial challenges, newer strategies involving bNAb therapy have shown promising results. Increasing the potency and half-life of bNAbs may result in a more durable viral suppression (48–50) and the combined use of bNAbs targeting non-overlapping epitopes have been shown to lead to longer-lasting control and prevention of the development of resistance (51–56). Additionally, manipulation of bNAb size to promote access to the virological synapse (53, 57) or the utilization of Abs that target CD4 or coreceptors may prevent viral entry and inhibit of cell-to-cell transmission (58, 59). Furthermore, recent studies have also shown that early and prolonged treatment with bNAbs may affect the size of cell-associated HIV reservoir (12, 60). However, the latent HIV reservoir is in a quiescent state and may not actively express viral antigens recognized by antibodies. Therefore, combinatorial approaches that involve bNAbs and latency reversal agents (LRAs) may be necessary to determine the ability of bNAbs to kill infected cells. In a humanized-mouse model of HIV, it was reported that the combination of LRAs and bNAbs interfered with the establishment and maintenance of the HIV reservoir (61). An additional study showed that the bNAb PGT121, together with a Toll-like receptor 7 (TLR7) agonist, delayed viral rebound in SHIV-infected monkeys, potentially due to a reduction in the viral reservoir (62). These data suggest that combination therapy utilizing bNAbs and LRAs may have utility in reducing the size of the reservoir HIV infected individuals.

In contrast to ART, bNAb therapy has also been shown to promote the emergence of HIV/SIV specific CD8 T cell immunity. There are several studies that suggest that bNAb therapy may help to induce CD8-specific responses and are also effective at delaying viral rebound during ATI (56, 60, 63, 64). However, the mechanism responsible for this observation has yet to be determined. Chronic Progressor (CP) CD8 T cell responses are not effective at controlling viral replication and of more than 30 clinical trials with therapeutic vaccination, none have led to sustained control of viral replication in a substantial number of subjects when ART was discontinued (16, 17). One study suggested that this may be due to the fact that the HIV-specific CD8 T cell responses that were generated were not as effective as those found in HIV controllers (65). Our data suggest that the combination of these enhanced but suboptimal CD8 T cell responses generated by therapeutic vaccination and CD4bs bNAbs could have synergistic antiviral effects. This may be particularly effective in shock and kill strategies when maximal eradication of infected CD4 T cells is needed during defined periods of latency reversal. Several clinical trials have shown that the combination of an LRA with either a therapeutic vaccine (66, 67), or bNAbs (68) have not led to extended remission in the majority of subjects. Our results suggest that a combination of bNAbs and therapeutic vaccination to enhance CD8 T cell responses may lead to better control after treatment with LRAs.

It should be noted that the effect of bNAbs *in vivo* may be mediated by the formation of immune complexes which subsequently leads to enhanced antigen uptake and presentation by antigen presenting cells. This indirect effect would not be seen in our system since we did not include any antigen presenting cells in our experiments. Our data do suggest that there is synergy between bNAbs and CD8 T cells, which was most clearly demonstrated with CD4bs bNAbs. Synergy has been observed with classes of antiretroviral drugs that inhibit HIV infection through independent mechanisms (45). The fact that bNAbs and CD8 T cells act independently to suppress HIV replication *in vitro* may potentially partially explain the observed synergy. However, the exact mechanisms responsible for this synergistic effect are unclear and warrant further investigation. Additionally, our study focused on CD8 T cells from a small cohort of HIV controllers. Interestingly, it appeared that ES CD8 T cells were more effective than VC CD8 T cells at controlling virus replication which is consistent with prior observations of differences in T cells from these two groups of controllers (19). It would be beneficial to determine if bNAb therapy can also enhance suppression by CP CD8 T cells. In a prior study we showed that ES CD8 T cells were more effective than CD8 T cells from viremic CP who were not on ART at suppressing virus replication in this assay (69). However, given the challenges of infecting CD4 T cells from CP on ART in the presence of residual intracellular antiretroviral drugs, we were unable to investigate whether synergy would also be seen in these patients. Finally, further *in vivo* studies are needed to determine whether these combined treatments will result in the reduction of the size of the HIV reservoir.

In this study, we have demonstrated that combination of bNAbs and CD8 T cells enhances suppression of HIV infection. These data suggest that this approach may be particularly effective in combination with LRAs and shock and kill strategies, when maximal eradication of infected CD4 T cells is needed. This approach should be considered in future therapeutic studies.

## Data Availability Statement

The datasets presented in this article are not readily available. Requests to access the datasets should be directed to Joel Blankson, jblanks@jhmi.edu.

## Ethics Statement

The studies involving human participants were reviewed and approved by Johns Hopkins University Institutional Review Board. The patients/participants provided their written informed consent to participate in this study.

## Author Contributions

RV performed the experiments and wrote the manuscript. CG performed the experiments. JBa and JBl supervised the experiments. All authors contributed to the article and approved the submitted version.

## Funding

This work was funded by Johns Hopkins University Center for AIDS Research (P30AI094189) and the NIAID (1R01AI120024).

## Conflict of Interest

The authors declare that the research was conducted in the absence of any commercial or financial relationships that could be construed as a potential conflict of interest.

## Publisher’s Note

All claims expressed in this article are solely those of the authors and do not necessarily represent those of their affiliated organizations, or those of the publisher, the editors and the reviewers. Any product that may be evaluated in this article, or claim that may be made by its manufacturer, is not guaranteed or endorsed by the publisher.
