# Supplementary material for: CD8 Effector T Cells Function Synergistically With Broadly Neutralizing Antibodies to Enhance Suppression of HIV Infection
Source: Front Immunol. 2021 Jul 29;12:708355. doi: 10.3389/fimmu.2021.708355 (PMC8358597; doi:10.3389/fimmu.2021.708355)
Supplement: Supplementary file 1 [file Presentation_1.pdf]

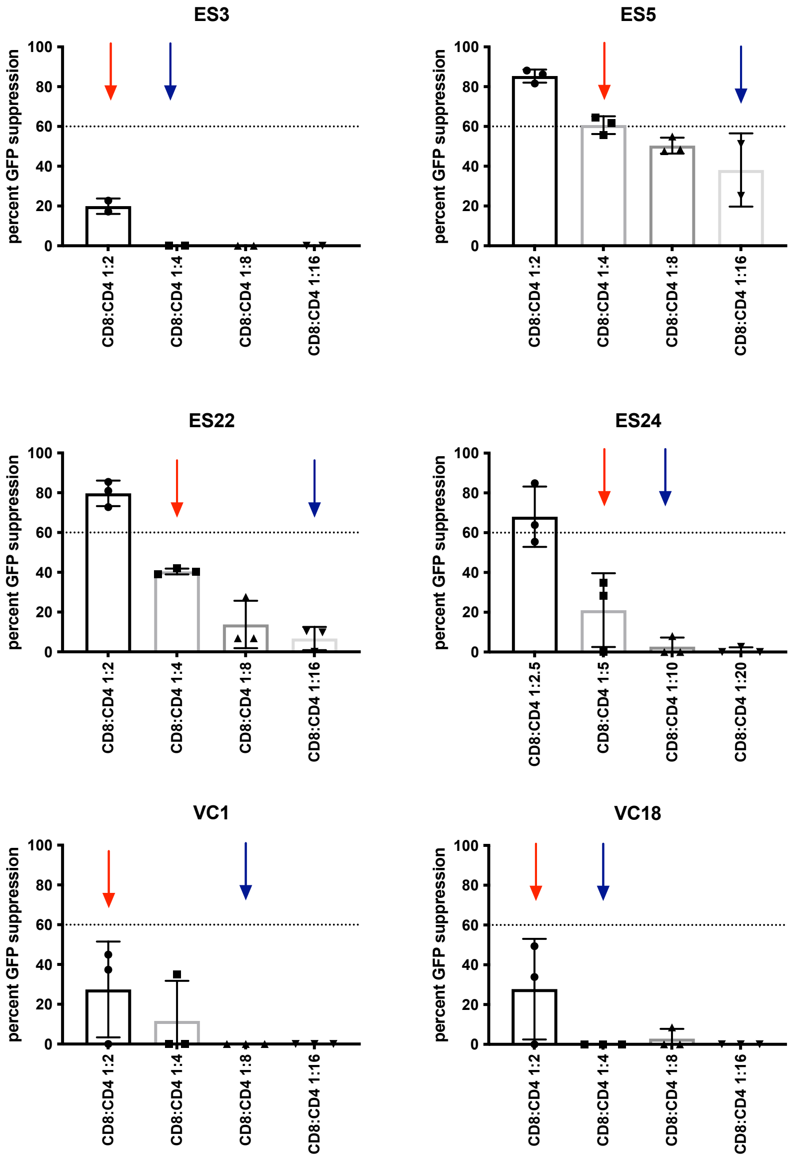

Supplemental Figure 1. IC60 and IC0 E:T ratios for each subject in study. Elite suppressors and viremic controllers isolated CD4 T cells were infected with 100ng of NL4.3deltaENV+X4 GFP virus per 100k cells without bNAbs and co-cultured with autologous CD8s at various E:T ratios. Each plot represents one subject, with multiple E:T ratios. The dotted horizontal line indicates 60% suppression, and the red arrow indicates the ratio for each subject that was closest to 60% suppression. The blue arrow indicates the lowest ratio or first ratio to show 0% suppression for each subject and was used for the low CD8 suppression analysis. Each point is a technical replicate, and the bar indicates the median.

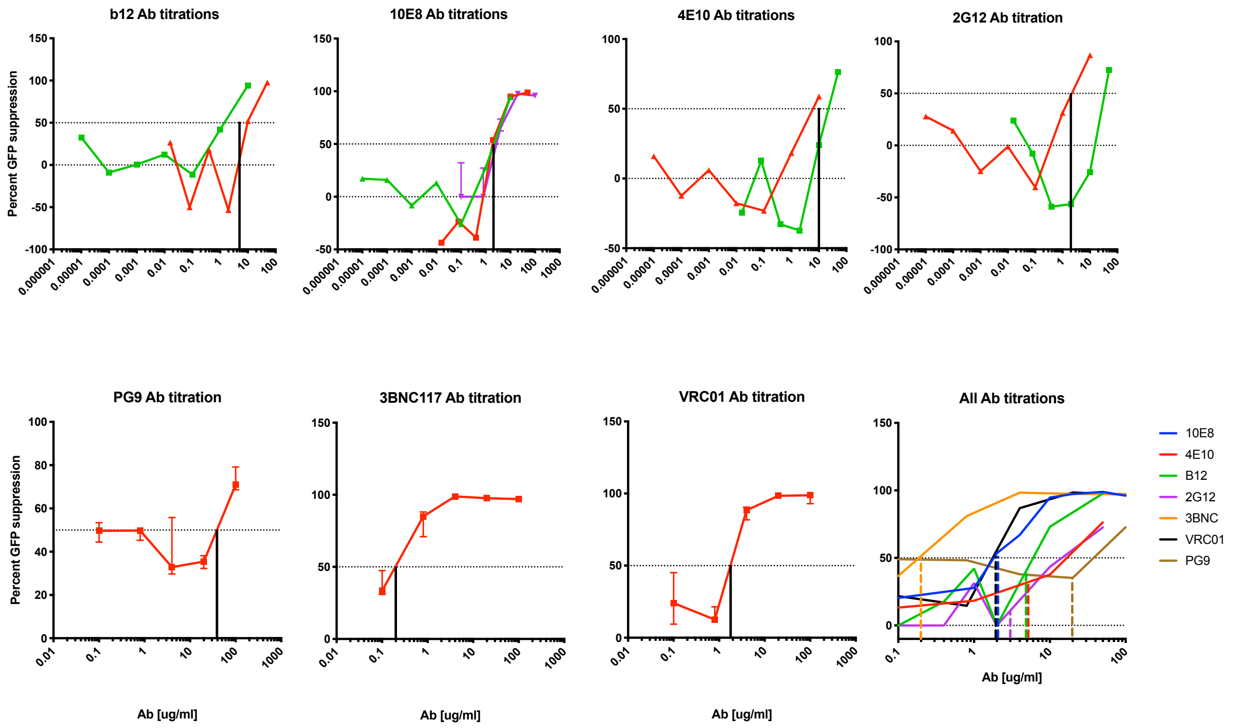

Supplemental Figure 2. IC50 bNAb titrations curves. Healthy donor (HD) CD4 T cells were isolated and infected with 100ng of NL4.3deltaENV+X4 GFP virus per 10ok cells that was previously incubated with a titration of a bNAb concentrations. (A) Each plot represents a titration curve of a particular bNAb to assess GFP suppression, more Ab present in the well results in more suppression. The presence of multiple-colored lines indicates that the Ab was titrated on more than one HD, the dotted horizontal line shows where 50% suppression meets the titration curve, the solid vertical lines marks the calculated IC50 concentration on the x axis. (B) This plot displays all bNAbs together, with vertical lines indicating the calculated IC50 for each bNAb. Data displayed are median values and 95% confidence intervals.

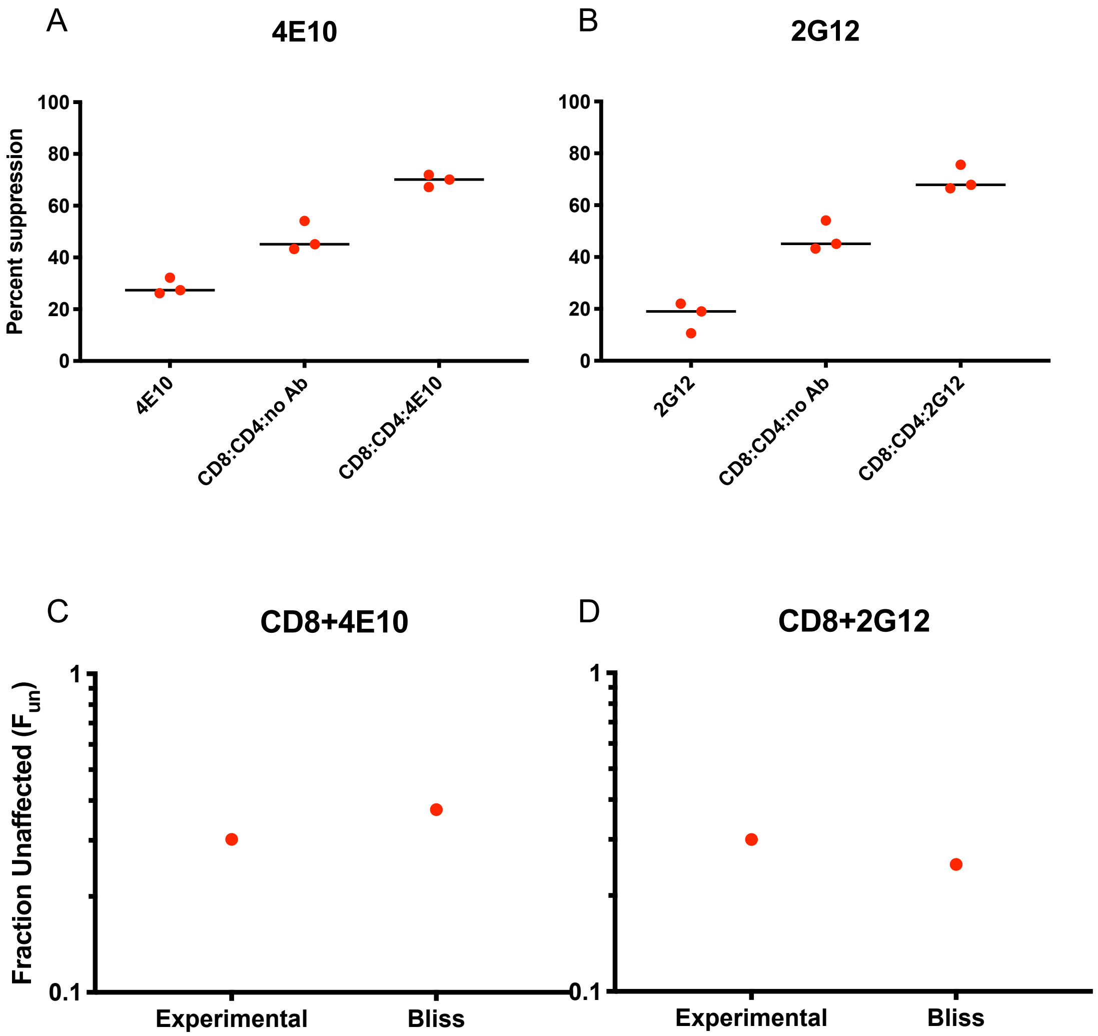

Supplemental Figure 3. 4E10 and 2G12 in combination with CD8s also result in enhanced GFP suppression. Due to limited availability CD8+bNAb suppression assays were completed with 4E10 and 2G12 using only one subject, ES3. (A) Displays the percent GFP suppression with each condition in isolation and combination, the addition of 4E10 (MPER) to autologous CD8 T cells lead to an enhancement of GFP suppression compared to bNAb or CD8 alone. The same trend was observed with 2G12 (V3, B). The Experimental Fun value calculated with 4E10 (C) and 2G12 (D) were compared to their predicated Bliss Fun values. The line indicates the median and each point represents a technical replicate within the assay. No statistical analyses were completed.

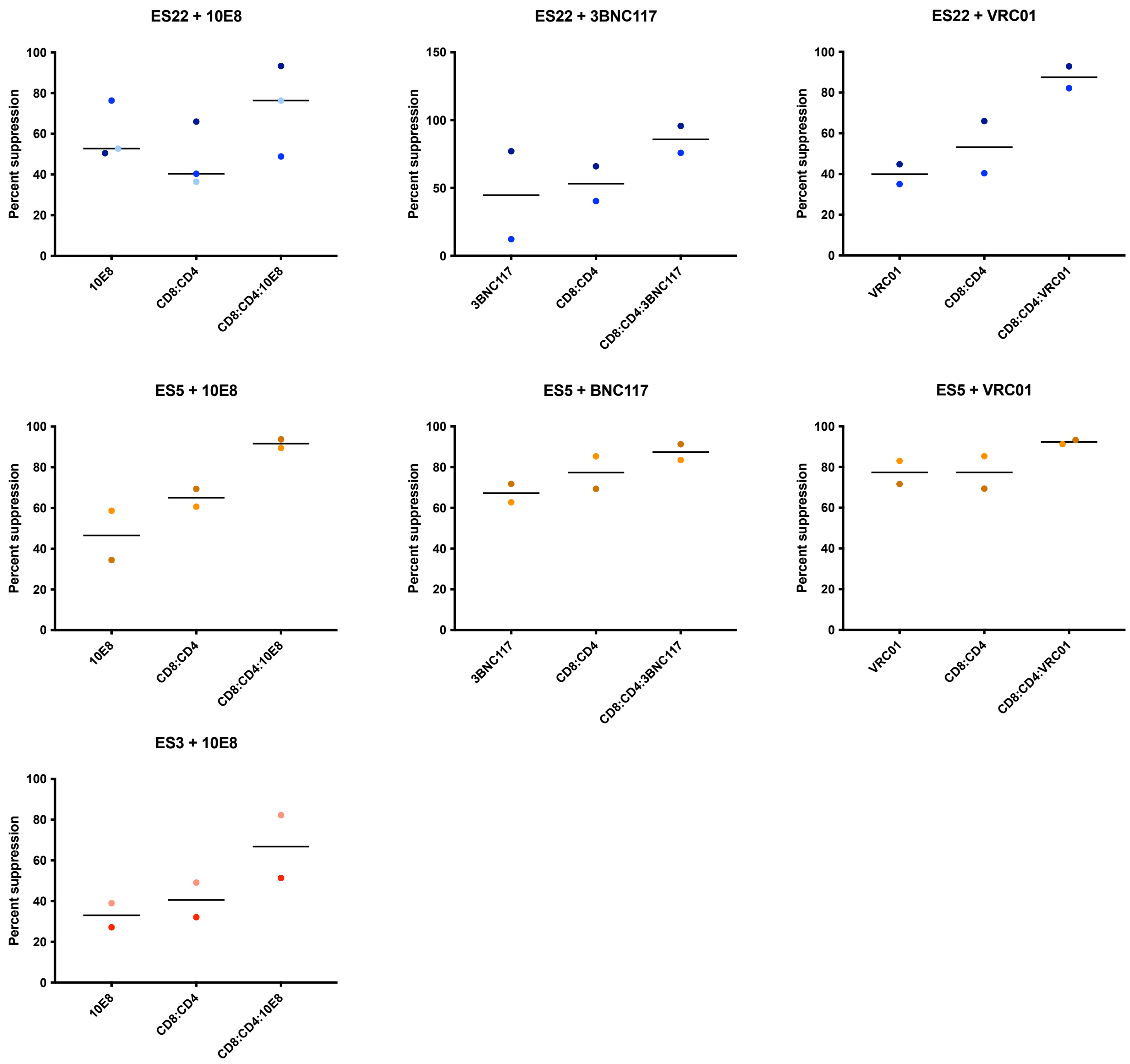

Supplemental Figure 4. Replicate experiments to determine variability of the assay. The CD8+bNAb assay was completed multiple times on select subjects to determine the repeatability of the assay. Each graph represents a subject and bNAb combination, and displays the conditions in isolation (bNAb only, CD8 only) and in combination (CD8+bNAb). Each point is an independent experiment and the calculated average of three technical replicates within the experiment. The line indicates median value.

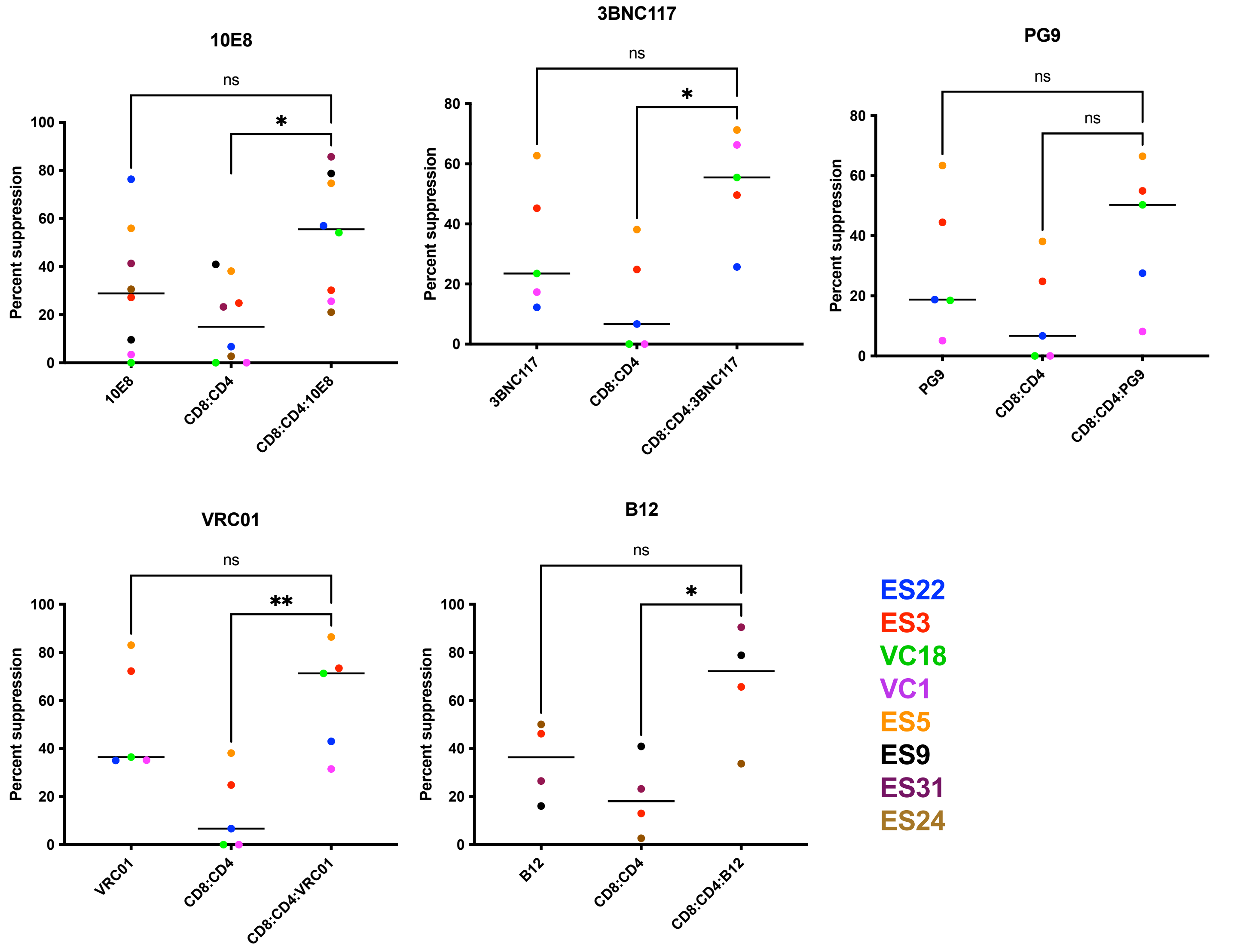

Supplemental Figure 5. Combination of bNAbs and autologous CD8 T cells at very low ratios do not enhance heterologous suppression. CD4 T cells from 6 ES and 2 VC were infected with GFP-NL4.3deltaEnv_X4 with and without bNAbIC50 and then co-cultured with multiple CD8:CD4 ratios. The first E:T which resulted 0% suppression or the lowest suppression available was used to compare the levels of suppression in bNAb only well, CD8 only and CD8+bNAb wells. The combination of any CD8IC0+bNAbIC50 lead to similar suppression of HIV infection compared to bNAbs alone. Each data point displays the average of three technical replicates performed for each condition. The line indicates the median, each colored point represents data from one subject, and the value is calculated by averaging three technical replicates within the experiment. Statistical analysis was performed using a 1-way ANOVA with Tukey's multiple comparison test, *p<0.05 and **p<0.05.

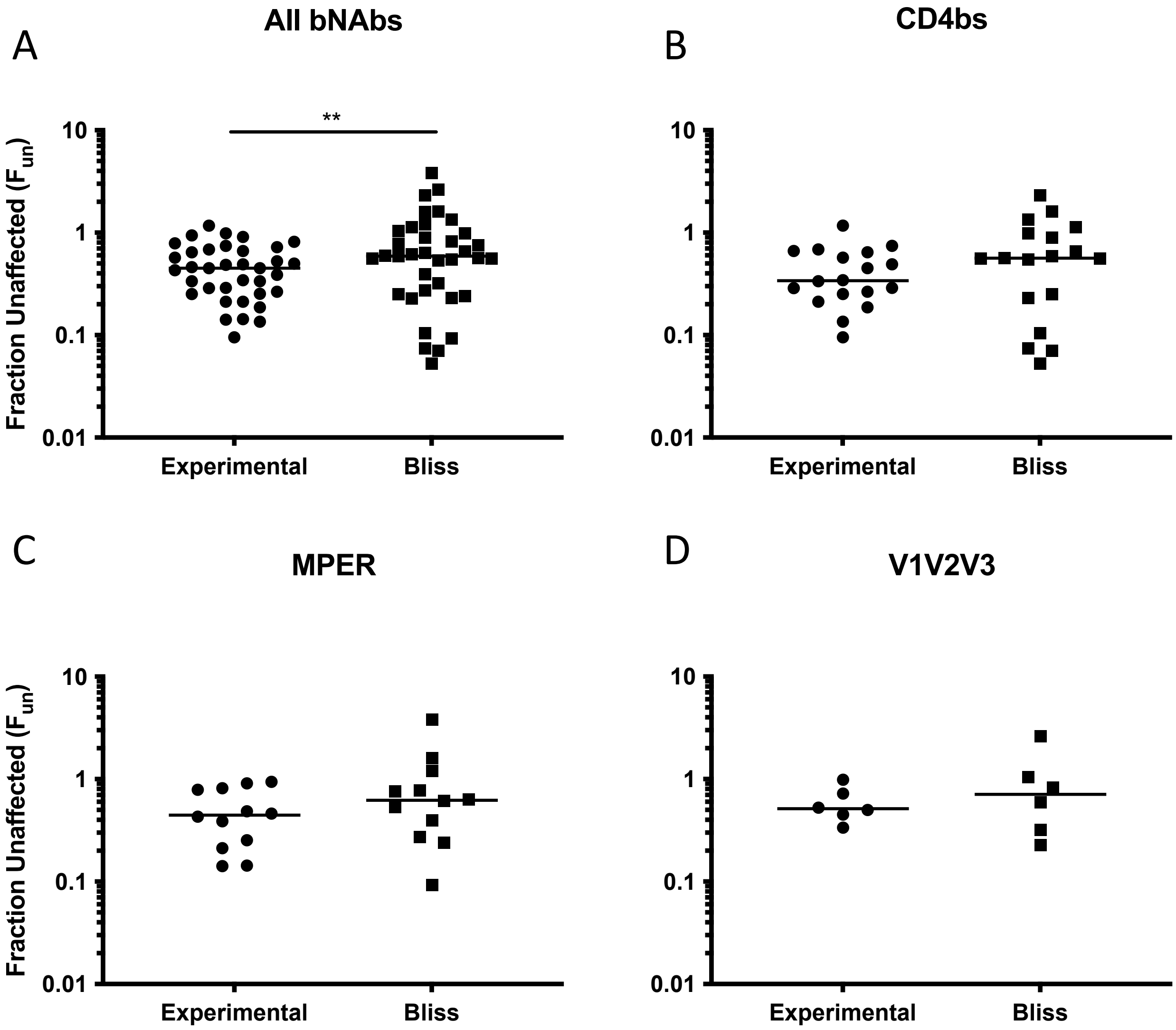

Supplemental Figure 6. Analysis of all bNAbs and CD8 T cells act synergistically to suppress HIV replication even at very low E:T ratios. Experimental F_{un} values from (A) all CD8+bNAb experiments, n=38, (B) all CD4bs CD8+bNAb experiments n=19, (C) all MPER CD8+bNAb experiments, n=13, and (D) all V1-V3 CD8+bNAb experiments, n= 6, were combined and compared to the predicted Bliss F_{un} values for the same conditions. Each BLISS or Fun data point is calculated from the average of three technical replicates performed for each condition. The line indicates median and each point indicates an independent experiment or predicated value. Statistical analysis was performed using paired t tests, **p=0.005.

Supplemental Table 1. Formulas and example calculations

Calculations	Formula	Examples
**% GFP suppression**	$100-[(\%GFP^{+}\ CD4_{(HIV+treatment)} \div \%GFP^{+}\ CD4_{HIVonly})*100]$	$\%GFP\ suppression_{HIV+CD8}$ $=100-[(\%GFP^{+}\ CD4_{HIV+CD8} \div \%GFP^{+}\ CD4_{HIVonly})*100]$<br>$\%GFP\ suppression_{HIV+10E8}$ $=100-[(\%GFP^{+}\ CD4_{HIV+10E8} *100) \div \%GFP^{+}\ CD4_{HIVonly}]$<br>$\%GFP\ suppression_{HIV+CD8+10E8}$ $=100-[(\%GFP^{+}\ CD4_{HIV+10E8}*100) \div \%GFP^{+}\ CD4_{HIVonly}]$
**Fraction unaffected (F_{un})**	$\%GFP^{+}\ CD4_{(HIV+treatment)} \div \%GFP^{+}\ CD4_{(HIVonly)}$	$F_{unCD8} = \%GFP^{+}\ CD4_{HIV+CD8} \div \%GFP^{+}\ CD4_{HIVonly}$<br>$F_{un10E8} = \%GFP^{+}\ CD4_{HIV+10E8} \div \%GFP^{+}\ CD4_{HIVonly}$<br>$F_{un+CD8+10E8} = \%GFP^{+}\ CD4_{HIV+CD8+10E8} \div \%GFP^{+}\ CD4_{HIVonly}$
**Bliss $F_{un(a+b)}$**	experimental $F_{un(a)}$ * experimental $F_{un(b)}$	Bliss $F_{un(CD8+10E8)}$= experimental $F_{un(CD8)}$ * experimental $F_{un(10E8)}$
**Independence**	BLISS F_{un} = experimental F_{un}	BLISS $F_{un(CD8+10E8)}$ = experimental $F_{un(CD8+10E8)}$
**Synergy**	BLISS F_{un} > experimental F_{un}	BLISS $F_{un(CD8+10E8)}$ > experimental $F_{un(CD8+10E8)}$
**Antagonism**	BLISS F_{un} < experimental F_{un}	BLISS $F_{un(CD8+10E8)}$ < experimental $F_{un(CD8+10E8)}$

Supplemental Table 2. bNAb calculated IC50s

bNAb	target	IC50 (ug/mL)	Subjects
**b12**	CD4 binding site	5	ES22 (2x), ES24, ES9, ES31, ES3,
**VRC01**	CD4 binding site	2	ES22 (2x), ES3, VC18, VC1, ES5
**3BNC117**	CD4 binding site	0.2-0.5	ES22 (2x), ES3, VC18, VC1, ES5 (2x)
**PG9**	V1V2 site	13	ES22, ES3, VC18, VC1, ES5
**2G12**	Glycan-V3 site	2	ES3
**10E8**	MPER	2-2.5	ES22 (3x), ES3, VC18, VC1, ES5, ES24, ES9, ES31
**4E10**	MPER	5-10	ES3

Supplemental Table 3. E:T ratios and mean % GFP expression per subject

Subject	CD8:CD4 ratio	%GFP suppression	Abs
**ES3**	1:2; 1:5	40.0	b12, VRC01, 3BNC117, PG9, 2G12, 10E8, 4E10
**ES5**	1:5	43.6	VRC01, 3BNC117 (2x), PG9, 10E8
**ES9**	1:20	41.0	b12, 10E8
**ES22**	1:2; 1:10	66.0	b12, VRC01 (2x), 3BNC117 (2x), PG9, 10E8 (3x)
**ES24**	1:5	21.1	b12, 10E8
**ES31**	1:10	51.6	b12, 10E8
**VC1**	1:2	3.5	VRC01, 3BNC117, PG9, 10E8
**VC18**	1:2	27.8	VRC01, 3BNC117, PG9, 10E8
